# Approaching Young University Students’ Suffering Following the Death of a Family Member: A Qualitative Study

**DOI:** 10.3390/healthcare14080991

**Published:** 2026-04-09

**Authors:** Cristobal Merino-Meza, María José Cáceres-Titos, Angela María Ortega-Galán, María Dolores Ruiz-Fernández, Jose Miguel Robles-Romero, E. Begoña Garcia-Navarro

**Affiliations:** 1 Health Sciences, University of Huelva, 21071 Huelva, Spain; pscristobalmm@gmail.com; 2 Independent Researcher, Concepción 3349001, Chile; 3 Department of Nursing, University of Huelva, 21071 Huelva, Spain; 4 Research Group ESEIS, Research Center COIDESO, University of Huelva, 21007 Huelva, Spain; 5 Department of Nursing, Physiotherapy and Medicine, University of Almería, 04120 Almería, Spain; 6 Facultad de Ciencias de la Salud, Universidad Autónoma de Chile, Temuco 4780000, Chile

**Keywords:** adolescent, grief, suffering, coping behaviour, family, palliative care, qualitative research

## Abstract

**Background/Objectives**: The death of a parent due to illness during adolescence constitutes a highly disruptive experience that compounds the developmental losses inherent to this stage of life. Distinguishing between the emotional and behavioural changes characteristic of adolescent development and those specific to grief can be complex, which may hinder the support provided by health, social care, and educational professionals. The aim of this study was to understand the grieving process and associated suffering in young university students who had lost a parent during adolescence. **Methods**: An exploratory qualitative design with a phenomenological approach was employed. Nine semi-structured interviews were conducted to examine in depth the experiences of suffering and grief associated with the loss of a family member among university students. The study adhered to the COREQ guidelines (Consolidated Criteria for Reporting Qualitative Research). **Results**: Among the main findings, the quality of the bond with the deceased parent emerged as particularly significant, as it influences the adolescent’s identity formation process. The loss of this parent may hinder processes of differentiation and independence, affecting the decisions young people must make as they transition into adulthood. This proves especially important in key life choices that shape their life project, such as vocational decisions and intimate partner relationships. **Conclusions**: Parental death during adolescence has long-lasting repercussions on identity construction and the shaping of one’s life project. It is necessary to strengthen psychosocial support within both clinical and educational contexts in order to address the specific needs of adolescents and young people undergoing this experience.

## 1. Introduction

Psychological distress among university students is a widely documented phenomenon and represents a growing concern in public health and higher education due to its impact on wellbeing, academic performance, and quality of life [1,2]. Among the various factors contributing to emotional distress in this population, the loss of a close family member emerges as a particularly significant experience, increasing vulnerability to mental health problems in later stages of life [3].

Various authors [4,5,6,7] have emphasised that suffering associated with experiences of loss not only represents an emotional burden but also shapes particular forms of coping and the development of resilience, especially when such experiences have not been adequately processed at the time they occur. The inability to integrate a significant loss, such as the death of a parent during adolescence, may have repercussions for the transition into adulthood—including the university stage—by influencing emotional regulation and perceptions of social support.

This suffering is not limited to cases of clinical bereavement; many students do not meet diagnostic criteria for a mental disorder, yet they experience significant distress that affects their quality of life, academic functioning, and social integration. Therefore, understanding grief from a perspective that encompasses the subjective meaning of suffering and adaptive coping strategies is essential in order to promote effective and context-sensitive interventions within university populations [8].

Adolescence is a stage of profound biological, cognitive, and psychosocial changes, during which individuals progressively construct their identity and redefine their interpersonal relationships [9]. Both classical and contemporary literature indicate that, although adolescents develop the cognitive capacity to understand death, their emotional experience is mediated by factors such as attachment, the quality of family relationships, and the social support available in their environment [9]. Parental death represents a profound threat to the adolescent’s sense of security and continuity, increasing the intensity of suffering.

To ensure analytical clarity in this study, it is necessary to conceptually differentiate three interrelated dimensions of the phenomenon. Grief is conceptualised as a dynamic and multidimensional process of adaptation to the perception of a significant loss [10], encompassing emotional, cognitive, behavioural, and socio-cultural response [11,12,13]. Suffering is defined as the specific subjective and lived experience of distress that arises when the individual’s perceived integrity is threatened by the loss [14]. Unlike grief, suffering is a holistic and existential state of severe distress where the self feels “broken” or challenged. Coping refers to the specific cognitive, emotional, and behavioural efforts directed at managing the internal and external demands of the grieving process [15].

This process is not linear, and its intensity and duration depend on multiple factors, including the nature of the death, the quality of attachment to the deceased, and individual coping strategies. Classical models, such as John Bowlby’s attachment theory [16], describe phases of reaction to loss that include shock, searching, disorganisation, and emotional reorganisation. Contemporary models, on the other hand, emphasise interindividual variability and the interaction between psychological, social, and cultural factors in the experience of grief [17]. From this theoretical perspective, grief in adolescence is inextricably linked to identity construction. During this stage, individuals define themselves in relation to other; thus, parental loss may act as a “biographical disruption” that forces a reconfiguration of the personal narrative and the sense of continuity. Furthermore, the processes of meaning-making and the continuity of symbolic bonds are key to understanding how students integrate the deceased parent into their future life project [18].

In the context of parental death from illnesses that allow for some degree of preparation, such as cancer, the grieving process includes both anticipatory grief—beginning with the terminal diagnosis—and grief following the death, which may manifest as deep sadness, sleep disturbances, difficulties in emotional regulation, and changes in social relationships [19,20]. Recent research suggests that in adolescents, prolonged or complicated grief is associated with an increased risk of affective disorders, generalised anxiety, and risky behaviours during university life [21,22]. Various studies [23,24] identify factors that modulate the grief experience: an emotional bond with the deceased parent—secure attachment is associated with more effective coping strategies; nature of the death—anticipated deaths due to prolonged illness create a double burden of suffering (diagnosis + grief); social and family support networks—the presence of emotional and community support predicts better adaptation; and emotional regulation and coping abilities—strategies such as actively seeking support and constructing a narrative of the loss are linked to better psychosocial outcomes [10,25]. Based on the research question, “How do university students experience and attribute meaning to the grieving process following the loss of parent during adolescence?”, the main objective of the present study was to understand the experience of the grieving process in university students who have lost a parent. Specifically, it aimed to explore the characteristics of the contexts and circumstances surrounding the parental loss, as well as describe the individual’s experience of the parent’s death during adolescence and its evolution over time.

Understanding the grief experience in these students would provide valuable information to address this issue more effectively, contributing to the prevention of mental health problems in adulthood. Moreover, exploring this phenomenon could help explain a significant portion of the suffering experienced by university students, considering the impact this process has on their academic, personal, and social development.

## 2. Materials and Methods

### 2.1. Design

An exploratory qualitative study with a phenomenological approach was designed. This approach aims to understand the phenomenon from the actor’s own perspective, capturing the subjective human experience in relation to the suffering caused by the death of a parent. It examines, in depth, the meaning attributed to this process by the participants themselves, as well as the strategies they employ to cope with it. The study follows the COREQ guidelines (Consolidated Criteria for Reporting Qualitative Research) [26] to ensure transparency and rigour throughout the research process.

### 2.2. Participants

The study participants were university students (aged 18 or older) who had experienced the death of a parent during their adolescence (between the ages of 10 or more), and whose parent had passed away more than four years prior, providing a retrospective account of their grieving process. An additional inclusion criterion was that the participant had lived with the parent before their death. Cases with a diagnosed mental health condition prior to the parent’s death were excluded.

A snowball sampling method [27] was employed. In phenomenological studies, the primary objective is to achieve a profound understanding of the lived experience; small sample sizes are common and methodologically appropriate, as demonstrated in similar studies [3,28,29,30,31]. Initially, participants were recruited through class representatives, with no professors present, to minimise selection bias. The final number of students included was determined according to the criteria of data saturation, when no new meaning units, codes, or thematic variations emerged in the final interviews. Data analysis was conducted concurrently with data collection, allowing the research team to identify redundancy in participants’ narratives [32]. The sample size was deemed adequate according to the concept of information power [33], which posits that the higher the specificity of the sample, the depth of the analysis, and the richness of the data, the fewer participants are required.

The study adhered to the international ethical principles established in the Declaration of Helsinki. All personal information collected was stored in accordance with legal requirements for the protection of personal data and digital rights (Organic Law 15/1999 of 13 December 1999 and Organic Law 3/2018 of 5 December 2018).

Due to the sensitive nature of the phenomenon under study, an emotional safety protocol was implemented. At the conclusion of each session, a debriefing process was conducted to allow participants to express their feelings and sensations following the interview. Furthermore, all students were provided with a comprehensive list of psychosocial support resources and bereavement units available within their university and local environments. It was pre-established that, should any risk of complicated grief or suicidal ideation be detected during the narrative, the research team—comprising mental health professionals—would facilitate an immediate referral to specialised services. However, it was not necessary to activate this procedure in any of the cases.

### 2.3. Data Collection

The study was conducted between February and June 2025. Initially, participants were recruited through class representatives, with no professors present, to minimise selection bias. A semi-structured phenomenological interview was employed as the primary data collection method. The interview guide was validated by experts in the field and initiated with an open-ended question: ‘Could you describe your experience since the death of your family member occurred?’. To explore the phenomenon in depth, follow-up and probing questions were used, such as ‘How did this event affect your daily life at the university?’ or ‘Which moments have been the most challenging for you during this process?’. Once it was confirmed that participants met the inclusion criteria, they were scheduled for an online semi-structured interview to provide detailed information on how they experienced such events [34]. Interviews did not exceed 80 min in duration.

To ensure validity and reliability, the coding and discourse analysis process was conducted independently by three members of the research team. Any discrepancies were discussed until a consensus was reached.

The final sample size was determined based on the principle of information power [35] considering the high specificity of the study’s aim and the quality of the dialogue obtained. Data collection and analysis were conducted through a concurrent and iterative process, allowing the research team to monitor for thematic redundancy.

### 2.4. Data Analysis

Documents were prepared and a hermeneutic unit was created using CAQDAS (computer-assisted qualitative data analysis software) ©Atlas.Ti Scientific Software, version 23 Development GmbH (Berlin, Germany), which included all transcribed documents used in the analysis. The interviews were analysed using the method proposed by Giorgi [35], consisting of three stages.

In the first stage, a thorough reading of the transcripts was conducted by two researchers. In the second stage, a further reading was undertaken to extract all units of meaning. These units were labelled and grouped according to initial codes (e.g., ‘shock’, ‘academic detachment’, and ‘symbolic legacy’). These codes acted as the primary link between the raw data and the descriptive subcategories. Through a constant comparative process, the research team refined these codes until a consensus was reached on the 10 final categories, ensuring that each category was rooted in the participants’ original expressions (phenomenological reduction).

In the third stage, following an interpretative process in group meetings, the categories were clustered into overarching themes based on their shared characteristics. Finally, interpretations of the content within each category were made according to the phenomenon or experience described.

Interpretative decisions were guided by the search for the essential structure of the bereavement phenomenon. During the transition from “meaning units” to the “psychological structure of the phenomenon,” the research team moved beyond descriptive coding to engage in a reflexive process regarding how each unit revealed a specific dimension of the student’s suffering. For instance, when identifying the category “ Immediate emotional impact and suspension of one’s own experience at the moment of loss”, the interpretative decision prioritised the lived experience of “unreality” over mere sadness, thereby capturing the phenomenological structure of adaptive denial described by participants.

The study acknowledges the positionality of the researchers—a team composed of nursing and psychology professionals with expertise in mental health and palliative care. While this clinical background facilitated empathy and depth during the interviews, it also necessitated a conscious exercise of epoché (bracketing) to avoid projecting established theoretical models of grief [10] onto the students’ lived narratives. Reflexivity was maintained through field diaries and regular team meetings, where the researchers’ emotional reactions to the accounts of loss were discussed. This ensured that interpretations remained strictly anchored in the participants’ voices rather than in prior clinical biases.

To ensure credibility, investigator triangulation was performed, and preliminary findings were presented to a subgroup of participants (member checking) to confirm that the identified categories faithfully reflected their lived experience. To ensure the scientific rigour of the study, triangulation was carried out among different participants and researchers, considering the researchers’ capacity for reflection and the relevance of the findings. Disagreements were resolved through constructive debate sessions and by returning to the original transcripts (data audit). The final consensus was reached by the ability of a category to holistically encompass the expressed subjective experience, thereby ensuring the internal consistency and credibility of the findings.

## 3. Results

The overall sample for the study comprised nine individuals, five women and four men, with a mean age of 20.44 years. The mean age at the time of the parent’s death ranged from 11 to 19 years, with five female and four male parents deceased. The participants’ sociodemographic characteristics are detailed in Table 1.

From the phenomenological analysis, ten categories emerged that describe the essential structure of the adolescent grief experience following the loss of a parent (Table 2).

Although these categories are described individually to ensure methodological rigour and transparency, they are interconnected through a narrative logic that reflects the student’s journey as a dynamic continuum. This journey is organised into three overarching phases that capture the global and transformative nature of the process: (1) The Existential Fracture (categories 1–2), representing the immediate impact of the loss and the disruption of the life project; (2) Navigating Vulnerability (categories 3–7), which encompasses the relational and academic challenges and renegotiation of bonds within the academic context; and (3) Ontological Reconfiguration (categories 8–10), describing the long-term integration of the loss into the student’s identity and future purpose. This structure allows for an exhaustive analysis of the phenomenon while maintaining a clear narrative of the grieving process as a global and transformative experience.

### 3.1. Anticipation of Death as a Process of Emotional Activation

During this period preceding the death, participants described a particular closeness to the parent, characterised by a more mindful attention to their personality, daily routines, and the nuances of their everyday relationship. The news of the illness and the imminence of death prompted a focus on the bond, whereby the adolescent began to see the parent as an individual, recognise their qualities, and share moments that took on special significance.

At this time, some participants reported being able to express affection, ask for forgiveness, or show love, experiencing this period as a space for emotional closure. In other cases, the lack of family communication regarding the seriousness of the illness hindered this process, subsequently generating greater confusion in the face of death.

“*In everyday life, you don’t stop to recall the whole process—in this case, the grieving process; you mainly remember the person rather than the process itself.*”

(I)

As an example of communication and family dynamic difficulties, this case reflects a conspiracy of silence prior to the parent’s death, beginning with the awareness of the impending death of the mother or father concerned:

“*And then I thought, they’re hiding something from me—I remember thinking, it can’t really be this bleak—and that’s when I spoke to the doctor, and he told me.*”

(III)

This preceding process triggers emotions and thoughts that shape a change or renewed emphasis in the quality of the bond, depending on how the student and the parent relate to one another during this time. Awareness of the parent’s possible mortality becomes news that fosters a particular closeness, or at least a shift in the relationship between them, accompanied by a heightened focus on the parent as a person.

This period unfolds as a kind of springtime in the relationship, ultimately leading to a form of emotional closure prior to the event of death:

“*And afterwards, once we knew, we tried to support my father in every way possible and make his illness easier for him.*”

(II)

“*I had to put on a brave face—she had to see us cheerful. Everyone kept saying, ‘You can’t cry; your mother needs to see you happy.*”

(III)

### 3.2. Immediate Emotional Impact and the Suspension of One’s Own Experience at the Moment of Loss

After the death, several participants described a sense of unreality in which, despite being aware of the death, they were unable to experience it as their own reality. This disbelief was reflected in the continuation of routines previously shared with the parent, such as waiting for their return or maintaining daily behaviours as if they were still present. It was the repeated absence in these familiar spaces that gradually allowed the death to settle as a real event in the adolescent’s experience.

“*I didn’t accept my father’s death at that moment… because I remember that I didn’t even cry when they told me he had died.*”

(II)

“*It may have helped me, because perhaps if I had accepted at that moment that my father was dead—given my age and the person I lost—I might have done, who knows, something reckless.*”

(IX)

In some cases, they described how their own emotions were overshadowed by the need to attend to what was happening around them.

“*But I saw my mother crying, I saw my aunt crying, and my feeling of sadness was more for them—it was a sense of putting myself in their place rather than focusing on what I was feeling myself.*”

(V)

“*Everyone was crying, and I tried to offer support, tried to lift everyone’s spirits. I worried more about others than about myself—that felt easier.*”

(III)

“*But because they saw me as so ‘normal’—I mean, I was just there—everyone was more concerned about those who were worse off, and at that moment I felt kind of in a cloud, neither happy nor sad.*”

(VIII)

The cultural rites associated with death are part of this experience, being experienced more as a sequence of events than as a fully conscious process.

“*The day after my father’s death, my best friend and his mother came to see me… my girlfriend also came… we were all at the funeral home together.*”

(V)

“*To the point that whenever I felt sad or sorrowful, I knew my father might be at the cemetery, but I would go there and talk to him—that brought me some relief.*”

(II)

### 3.3. Understanding Death and Loss

After the death, the loss is not perceived as a single event but as an experience that extends over time. Participants described a sense that the absence remains present in their daily lives. Understanding of the death develops gradually, giving the experience meanings that evolve over time. Feelings of loyalty toward the deceased parent emerge, and in some cases, a sense of abandonment associated with the physical absence.

This category includes statements that highlight the unexpected nature of the event or that reflect some degree of processing regarding this enduring aspect of the loss.

“*Moreover, my father died young—he was only 43 years old.*”

(II)

“*I mean, I would never have imagined it. I thought, I don’t know, that he would live to see other experiences, that he’d be around until I grew up, got married, and had children.*”

(III)

### 3.4. The Internal Phenomenology of Loss

Efforts to make sense of or assign meaning to the parent’s death do not necessarily develop through a logical or rigorous approach. Rather, they emerge from particular situations or experiences available within the adolescent’s context and converge into an explanation that provides relief, appearing as a personal elaboration or the adoption of a meaning drawn from the surrounding cultural or familial framework. More than a mere succession of emotions, the internal phenomenology of loss reveals a constant effort to maintain continuity of the bond through ‘signs’ or academic promises. Meaning units such as ‘waiting for the return’ or a ‘void in the chest’ are not merely symptoms of grief; they are manifestations of a suspended reality where the adolescent oscillates between adaptive denial and the integration of absence as a new personal truth.

“*After a few months had passed since his death, it was as if he were away on a trip rather than having died—I was always expecting him to come back.*”

(II)

“*So, in a way, there were signs—if you look at it from a religious perspective—there were signs of what was going to happen, and that’s why I clung to them.*”

(V)

These meanings or interpretations are often accompanied by emotional commitments or loyalties toward the deceased parent, or toward certain qualities or characteristics of the parent that the bereaved highlights.

“*Also a promise—well, that helped me with my degree too. I mean, I promised him I would complete it.*”

(VI)

In the participants’ accounts, it is possible to observe how they distinguish a series of emotional reactions and activations at different moments following the parent’s death. These can be understood as a phenomenology of loss, encompassing the common feelings in response to the death of a loved one. Among these, various sensations emerge, including abandonment, helplessness, loneliness, longing, disbelief, and confusion, among others.

The experience of loss emerges as a biographical disruption that alters not only the family structure but also the frames of reference through which the adolescent constructs their future. The narratives suggest that the parent’s death acts as a ‘breaking point’ where the previous life project (e.g., ‘staying with my parents forever’) collapses, forcing a mandatory re-signification of autonomy. This transition is not merely chronological; it is an identity crisis where the young person must suddenly ‘become an adult’ to fill role gaps and expectations.

“*Looking back, that was, well, strange—a somewhat unusual reaction for me. Later, I don’t know, I joked about it; let’s say the first few hours were more intense.*”

(III)

“*After a few months had passed since his death, it was as if… maybe he—I experienced it as if he were away on a trip rather than having passed away; I was always expecting him to come back.*”

(II)

“*A void, a void because, for many years, I kept hoping he would come back, and he never did. So it was a void—he took, you could say, the best part of me with him.*”

(I)

“*Let’s say it overwhelmed me—I kind of became detached, as if I had transformed reality into truth; even my behaviour was somewhat out of step.*”

(III)

### 3.5. Changes in Life Perception

Participants reported that this experience brought about a change in the way they understand life. The loss acts as a turning point, altering their perception of the world and fostering processes of personal re-signification. This change is also reflected in adjustments to their life plans and in the acceptance of transformations within their family and social environment. This synchronic disconnection highlights how bereavement strips the individual of their sense of belonging to the social collective, placing them in an existential periphery.

“*And I don’t know, I remember once going to the bathroom to get my clothes, and everything felt so quiet—the house hadn’t been this quiet when my mother was around, as far as I remember… and that’s when I really started to miss her, in the sense of realising that this was actually happening.*”

(III)

These events are identified and used by the student as a way of providing access to the origins of their thoughts and to the meanings they attribute to the loss during the interview situation.

“*I didn’t want to do anything in life, not even work. The thing is, all the expectations were placed on my sister, so I thought I would be the one to stay with our parents forever, while my sister would be the one to become someone.*”

(I)

Furthermore, some of these experiences provide a link between one’s own reasoning and the realm of reality, appearing to support personal understandings and establishing a sense of personal truth regarding certain conclusions. Within the narrative, these elements function as certainties that shape how personal reality is assumed, acting as points of reference between meaning-making processes and the reality being confronted.

“*I always, even when I was older like now, looked forward to Saturdays. But gradually, little by little, I got used to the fact that he was no longer there.*”

(II)

“*So, one of those people came into my life and gave me a couple of messages that I couldn’t understand at the time… ‘you have to go through a period of sadness first,’ and I didn’t know that. Later, they explained to me that the hardest moments are always the deaths… and that’s why I clung to that so much, because there were many signs showing me that it would all happen that way.*”

(V)

“*But now I’ve accepted that he passed away. And that my mother has every right to rebuild her life and everything, because at the time I couldn’t accept that either.*”

(II)

In this way, it can be seen how participants begin with a process of denial rooted in reality, which they gradually overcome, eventually accepting the loss. The needs in their emotional bond with the deceased parent start to be fulfilled through others, recognising that life goes on—not only for themselves but also for their closest loved ones.

### 3.6. Reconfiguring Family and Social Relationships After Loss

Following the loss, interpersonal relationships—both within the immediate family and with the extended family, friends, or others—undergo significant changes as a result of the parent’s death. There is a relational dissonance between the bereaved and their peer group. While the university promotes a stage of expansion and vitality, the grieving student experiences a premature ‘emotional aging.’ This communicative gap with peers reinforces isolation—not due to a lack of social presence, but because of the environment’s inability to sustain a narrative of finitude within a context of youth.

“*I always had a lot of trust with my father, to the point that after he died, one of my mother’s sisters came to live with us… And I, looking for a refuge because I couldn’t share things with my mother, started telling her my things.*”

(II)

These changes occurred primarily at the level of the emotional bond between the bereaved and other family members, and could take the form of either distancing or closeness, shifts in attitudes toward others, or changes in the routines and roles carried out by both close family members and the bereaved themselves.

“*I was kind of angry—I don’t know, maybe I was angry with women, or I got angry because… my father was gone.*”

(II)

Moreover, differences can be observed in the types of relationships in which these changes occur (sibling, parental, and so on).

“*And that’s when I started to form a much closer bond with her (my sister), because before, our relationship had been quite bad.*”

(IV)

“*I felt a lot of anger toward her because she didn’t show me the affection I needed, especially after my father passed away.*”

(II)

Sometimes these changes did not only occur within the specific bond with a particular family member but also manifested more broadly in family dynamics, routines, and overall organisation.

Participants also frequently described changes in their attitudes toward new friendships or relationships outside the family, showing some degree of contrast compared with the period before or after the parent’s death.

The daily paralysis reported by students should not be understood merely as physical lethargy, but as a fracture of biographical temporality. The parent’s death halts the ‘project time’ (the university), confronting the student with a suspended present where academic goals lose their meaning in the face of the urgency of the affective void.

“*My father passed away, and a distancing occurred with my father’s entire family.*”

(II)

“*I compare myself to how I was before. Now at university, new classmates arrive, and I’m like this (makes a gesture of indifference)—it’s like, no, I’m not interested in connecting with anyone now, and it wasn’t like that before.*”

(I)

The experience of loss influences the way participants perceive themselves during this stage of life. They describe processes of increased autonomy, moments of rebellion, and an evolution in how they relate to others. Empathy and the pursuit of emotional intimacy emerge as traits that are strengthened following the experience of grief.

“*It was, it was when I was about 14—it felt like a hole in my chest that nothing could fill. And I kept hoping, hoping that at some point it would fill, not with someone else, but with the same person.*”

(II)

“*Both its positive and negative sides, I think, helped me. Negative in the sense that during my adolescence… it led me to develop a very tough, resistant attitude toward the world.*”

(IV)

“*I missed him; there were so many things I needed to talk to him about. I had just turned 14, so much was happening, and I was just starting secondary school.*”

(II)

“*I went through a difficult time in my life and chose that escape, so to speak, by smoking. Before turning to something more harmful in the short term, I opted for something with long-term effects.*”

(IV)

“*I even went so far as to question my faith, and this is the first time I’ve ever said that.*”

(IX)

“*To show that we were mature people, and this made us grow up—you could say it was suddenly the test we needed.*”

(III)

### 3.7. Impact on the Construction of the Self During Adolescence

There is evidence of the development of empathy and an interest in understanding others on an individual level, demonstrating awareness of needs and processes that occur beyond oneself—the individualisation of the other.

“*…She was more rebellious and sometimes liked to go against the grain. I don’t know, if my mother wanted to give her advice and it didn’t align with her ideals, she could be distant at times—back then, she was more detached.*”

(III)

“*I have the case of another friend, who is quite immature as a person, but I’ve seen him, and I think that as a man or as a father, if he were one someday, he would be quite responsible.*”

(V)

The impact on the quality of interactions with others is also included, as highlighted in the following quotes.

“*They realised that even at my age, my father was always present—I always talked about him, always shared stories about him with my friends—and they gradually helped me accept that my father was no longer there, that he was dead and not alive.*”

(II)

In some cases, a more subtle type of change was observed, involving modifications in the capacity for emotional intimacy or basic trust toward others.

“*I was completely closed off to showing affection to others—I didn’t even show affection to my siblings, not even to my mother.*”

(II)

“*I think the fact that my mother passed away, in one way or another, caused me not to want to trust anyone, and not to feel that anyone was completely loyal enough for me to truly be myself.*”

(IV)

### 3.8. Memories and Connection with the Deceased Parent

Participants described their relationship with the deceased parent, referring to the meaningful routines they normally shared. They expressed the quality of the bond, as well as the character and qualities with which the parent was perceived, sometimes defining what the parent represented in a particular concept or idea.

“*The first thing was waiting for him to arrive—we always knew he would get there at a certain time, and we waited for him to get off the bus. When Saturday came, I would run to hug him—always on Saturdays, he always did the same, and I would run and hug him.*”

(II)

“*My father was not only my father; he was also my friend.*”

(VII)

These descriptions provided by the participants reflected, at a given moment, a particular closeness or special interest toward the parent during their illness.

“*I didn’t want to be alone either, so I started getting closer to him. We would stay up at night, watch things on TV together, talk about what was happening to me and to him. In a way, we tried to understand each other more, to develop a closeness we hadn’t had before.*”

(V)

“*I started, in a way, to rely on her as a mother during that process, from the moment she was diagnosed with cancer.*”

(I)

Exploration across the different interviews reveals how the figure of the parent is organised with a very personal quality, and does not in any way constitute a standard defined by the role. Instead, each individual emphasises the relationship with a particular quality and emotional content—the quality of the bond.

“*It was an incredibly strong friendship, where I could rely on him at any moment. If I felt sad, he was there; if I was happy, he was there; if I wanted to play something, he was there—for anything, anything I could think of, he was always there.*”

(II)

At times, participants described the moments and activities they shared with the parent, recounting them differently from other relationships within the family unit, turning these routines into markers of closeness between them.

“*I was the only one—I would go out, hug him, throw myself on him, and then he would carry me home in his arms.*”

(II)

“*Maybe my father and I didn’t spend the whole day together, but there would come a time in the afternoon when we would sit and talk alone.*”

(V)

Perceptions of the parent also sometimes included efforts to integrate negative aspects or flaws in their personality.

“*My father wouldn’t back down; he would get angry with me and stop speaking to me, and that used to upset me a lot.*”

(II)

“*Looking at it more objectively, my mother was always very concerned that we lacked nothing materially, but in terms of worrying about how I was—like, ‘Daughter, what’s wrong?’—no, it wasn’t like that.*”

(I)

### 3.9. Family and Cultural Worldview Framing the Experience of Death

The experience of grief is influenced by family values, religious beliefs, and cultural rites surrounding death. These elements shape the framework through which participants interpret and experience the loss. Frequently, the accounts included explanations or referenced information related to the values that dominated the family’s worldview.

“*We were really close, because there are four of us—my father, my mother, my sister, and me—so we were very united… if something happened to one of us, it affected everyone; it hurt everyone.*”

(I)

“*He taught me how to value things. Well, we’ve never been a family with much money, and through his life experiences and examples, he helped me understand and appreciate the things I have.*”

(V)

Of particular interest are the accounts related to religious practices or beliefs upheld by a family member or shared by the whole family, which shaped important aspects of the experience of loss.

“*At church, we would sing together—that’s where I got my gift for singing. My mother sang very well.*”

(I)

Also included are accounts referring to rituals related to death, typically concerning how the wake and funeral were conducted.

“*Well, I remember the preparations beforehand—the funeral home and the church. It was an evangelical church near the house where I lived. They were very kind; it was very beautiful.*”

(II)

### 3.10. Re-Signification and Life Lessons Derived from the Loss

Analysis of the accounts reveals an interest in drawing personal life lessons from the experience of loss, shaped by the figure of the deceased parent. Students highlighted virtues they identified in their parent, which primarily enabled them to engage with the social world in a way defined by this life event. This even fostered an intentionality to structure their future based on these lessons, for example by defining vocational interests, as illustrated in the following quotes:

“*I chose nursing because it really caught my attention, and even though I wasn’t very interested in health at first, I learned to love the degree. But it was my choice—it was what I decided for myself.*”

(IX)

“*I think it was mostly the path I ended up taking, yes, trying to find meaning in things I hadn’t seen before, but I believe that motivated me to study nursing.*”

(IV)

These conclusions often reinforced the idea of valuing close relationships, highlighting the importance of expressing oneself and understanding others, marked by interest and engagement.

“*Seeing life from a different perspective, learning to appreciate what we have, looking out for each other—those were things we either did very poorly before or simply didn’t have, and now we do.*”

(III)

“*We could say that what happened led to this outcome—that right now, we are a somewhat closer family.*”

(V)

“*My mother has always mattered to me, but I gave her a greater place in my life.*”

(V)

Similarly, there are accounts highlighting the importance of developing certain social practices or values, integrated as personal qualities that shape a way of maintaining a connection with the deceased parent, which are internalised by the adolescent.

“*More tolerant with people—now, if I feel bad, I show it and let others ask me about it.*”

(VII)

Taken together, these categories and subcategories show that grief following the loss of a parent is a complex and profoundly transformative experience, spanning time, relationships, and personal identity. The experience is not limited to the moment of death; it begins with the anticipation of loss and extends into a constant presence of absence, influencing the way young people understand life, relate to others, and perceive themselves. In this way, the phenomenon of grief emerges as a process that reshapes the student’s inner and relational world, leaving a lasting mark on their life trajectory.

## 4. Discussion

The results of this study provide an in-depth understanding of how university students experience the loss of a parent, highlighting a grief experience that unfolds across temporal, emotional, relational, and self-construction dimensions. This complex and multifaceted experience aligns with recent research emphasising the intensity and persistence of grief symptoms in young people following a significant family loss [36,37].

Our findings regarding the period preceding death reflect processes consistent with anticipatory grief, where the awareness of an imminent end prompts a relational reorganisation. This period, often experienced as a time of “mindful closeness”, allows the adolescent to recognise the parent as individual, facilitating—or in cases of poor family communication—a meaningful emotional farewell [38].

From a theoretical perspective, the disorientation reported by students can be interpreted through Attachment Theory [16]. The death of a parent represents the loss of a “secure base”, triggering anxious or avoidant attachment strategies within the university environment. We argue that the reported difficulty in concentrating is not merely a cognitive deficit but a manifestation of hypervigilance, a constant internal search for the lost attachment figure that depletes the emotional and cognitive resources necessary for academic success.

While previous research suggests that grief in early youth tends toward a search for independence, our findings reveal a paradoxical tension that challenges linear developmental models. Participants manifested a “forced autonomy” that originates not from maturity but from a sense of unprotectedness. This coexistence of external hyper-autonomy and internal fragility contradicts models that present resilience solely as a capacity for “overcoming”. Instead, our data suggests that autonomy may act as an adaptive mask concealing unresolved vulnerability. This is a crucial finding for higher education institutions; academic success and apparent maturity should not necessarily be interpreted as a resolution of grief but rather as a “survival functionality” that requires specific clinical vigilance from university health services [6].

Recent research has also documented that young people who have lost a close family member exhibit a high prevalence of prolonged or complicated grief, with implications for mental health such as persistent anxiety and sadness. A recent meta-analysis [39] found that 48% of young respondents reported intense and prolonged grief symptoms following a cancer-related loss, exceeding rates observed for other causes of death and reflecting the unique emotional burden of deaths due to prolonged illness. This corresponds with the intensity of emotions and the persistent sense of absence described by participants in this study, where death is not experienced as a punctual event but as a continuous presence that must be integrated into the adolescent’s life narrative.

Similarly, relational and familial role changes observed in the analysis—such as affective distancing, modifications in routines, and reconfigurations of intimacy—are consistent with studies showing that grief alters family dynamics and support relationships, affecting both extended family and peer connections [39]. Investigating these relational transformations in adolescents is essential, as the quality of social and family support has been associated with grief resolution and young people’s ability to adapt to social demands following the loss [40].

The findings suggest that parental loss during adolescence functions as a biographical disruption [40], where the expected trajectory of life is abruptly severed. This disruption forces the student into what Meleis [41] identifies as a complex transition that is both situational and developmental. The “turning point” identified by participants represents a qualitative shift in their relational ontology, often requiring a “premature mastery” of coping roles. The adolescent often abandons the developmental moratorium of student life to assume adult responsibilities, a process we theorise as a forced acceleration of the life cycle [42].

This transition intervenes directly in identity construction. Contrary to traditional models emphasising “detachment”, our results align with Continuing Bonds [18] and Meaning Reconstruction [43]. Participants maintained an active relationship with the deceased through internal rituals and the internalisation of their values. This symbolic continuity is not pathological; it is an adaptive mechanism that allows students to rebuild a belief system where finitude and vulnerability are integrated into a more resilient self-narrative.

From a clinical perspective, these findings underscore the importance of trauma-informed support in academic settings. The “daily paralysis” reported by students is not merely lethargy but a fracture of biographical temporality, where academic goals lose their meaning against the urgency of the affective void.

This study contributes to the literature by reframing parental loss during youth as a profound identity reconfiguration rather than a punctual emotional event. Recognising the importance of symbolic continuity and the subjective experience of suffering—aspects still underexplored in university populations—is essential for developing psychosocial supports tailored to the specific developmental needs of grieving students.

## 5. Conclusions

The results of this study provide an in-depth understanding of the experience of losing a parent during adolescence from the perspective of those who have lived it, identifying its emotional, relational, developmental, and sociocultural components. The phenomenological methodology employed facilitated direct access to the participants’ subjective experience, allowing for a description of the impact of the loss over time—from the stage preceding the illness to the current situation—across personal, family, and social domains involved in this process.

One of the most relevant findings shows that, in the period prior to the death, an affective closeness to the parent emerges, contrasting with the normative adolescent tendency toward differentiation. This movement of approximation generates an interest in knowing and internalising the parent’s qualities, forming a way to maintain the bond after death. This process overlaps with the developmental task of identity formation, creating a particular tension between non-normative grief and the demands of adolescence, with consequences for the young person’s future.

Additionally, it is observed that during the period of terminal illness, the adolescent adopts a rational and empathetic attitude focused on the needs of the parent and family, displacing their own emotional needs. This emotional containment breaks at the moment of death, giving way to reactions of disbelief and denial, evidencing the coexistence of two grief processes: the normative, typical of the developmental stage, and the non-normative, associated with parental loss. This phenomenon highlights the clinical importance of understanding the defensive mechanisms involved at this stage to prevent later complications.

The findings also show how the developmental tasks of adolescence, particularly peer group belonging, play a fundamental role in grief processing. The need to not feel different from others leads the adolescent to find in new relationships a space to recover experiential components of the lost bond. This suggests that overcoming grief is not limited to internal reorganisation but relies significantly on external relational experiences.

A key aspect identified is the phenomenon of “affirmation of reality” through significant events, which allows the adolescent to move from the unreality of the loss toward acceptance of the death as part of their personal history. Although this process is present in all participants, the timing varies, highlighting the uniqueness of each grief experience.

The quality of the bond with the deceased parent emerges as a central axis for understanding the meaning of the loss and the particular coping strategies employed. Loyalties, commitments, and the need to maintain continuity of the bond influence multiple areas of the adolescent’s life, including vocational choices, relationships with others, and identity development. This finding has important clinical implications, as exploring the nature of this bond can guide therapeutic interventions tailored to the uniqueness of each grief experience.

The experience of loss also accelerates processes of autonomy and responsibility, placing the adolescent in a supportive role toward other family members at a time when they themselves require emotional support. This role inversion later translates into difficulties in forming intimate relationships, characterised by greater emotional distance, heightened responsibility, and limitations in emotional expression.

Overall, these findings show that parental loss during adolescence is not only a painful event but also a phenomenon that interacts critically with the developmental tasks of this stage, reshaping identity, relationships, and the adolescent’s position in the world. Understanding this complexity is essential for designing clinical, educational, and family interventions that are sensitive to the specific needs of these young people.

### 5.1. Limitations

During the course of this study, several limitations related to the investigation of the phenomenon were identified. First, the study’s retrospective design introduces an inherent recall bias, as participants were required to reminisce about events that occurred, in some cases, more than four years prior. These accounts are inevitably mediated by the students’ current maturity and evolved cognitive capacity. While this implies that the narratives reflect a re-signification process rather than the raw intensity of the acute grief phase, this perspective also constitutes a significant conceptual strength. It allows for the observation of the long-term impact of loss on identity construction and the reconfiguration of life projects, offering a longitudinal depth that immediate interventions often lack.

From a methodological and ethical standpoint, the nature of parental loss during adolescence precluded a direct examination of the phenomenon during its most acute stage. Although clinical case analysis could have served as an alternative, our findings indicate that a significant number of adolescents do not seek formal psychological treatment following a loss. Therefore, while the delay in data collection may affect the immediate descriptive depth of the trauma, the university setting provides a unique and necessary window into the subjective experience of “non-clinical” suffering that might otherwise remain invisible to researchers and practitioners.

Furthermore, the transferability of these results is constrained by the geographical and cultural homogeneity of the sample. Grief is a construct deeply embedded in specific social norms and cultural rituals; thus, these findings must be interpreted within the participants’ specific socio-cultural framework. Caution should be exercised when attempting to apply these conclusions universally to diverse ethnic groups or different belief systems. The phenomenological saturation achieved in this study ensures depth within this specific context but does not claim to represent the vast global diversity of the grieving experience.

Finally, it is essential to recognise a potential selection bias inherent in the study population. The sample consists exclusively of university students, representing a trajectory of “relative success.” The fact that these young individuals have remained within the higher education system despite their loss suggests the presence of robust coping mechanisms, socioeconomic resources, or stable support networks. This “natural selection” may bias the findings toward a more resilient representation of grief. Consequently, the results may overshadow the experiences of more vulnerable youth whose biographical disruption led to a total collapse of their academic and personal projects, resulting in their departure from the educational system.

### 5.2. Implications in the Clinical Practice

This study is highly relevant to the fields of health and nursing as it addresses a life-altering experience—the death of a family member—during youth, a critical developmental stage in which emotional, behavioural, and coping patterns are shaped and may have lasting effects on health trajectories. Grief-related suffering among young people constitutes a significant psychosocial determinant of health that remains underexplored, particularly from a qualitative, experience-centred perspective.

From a nursing standpoint, understanding how young people perceive, express, and manage suffering following a family loss is essential for the development of sensitive, person-centred, and humanised care interventions. Nurses often serve as frontline professionals in community, educational, and clinical settings, where they encounter young individuals experiencing grief that may go unrecognised or be inadequately addressed.

By providing in-depth qualitative evidence, this study gives visibility to forms of suffering that are frequently normalised or silenced, offering valuable insights for the early identification of complicated grief, the prevention of mental health problems, and the strengthening of holistic nursing practices. Moreover, this research contributes to the advancement of nursing knowledge by deepening understanding of the subjective dimensions of care, highlighting the importance of qualitative inquiry as a core methodological approach for improving clinical practice, professional education, and the design of health interventions aimed at young populations.

## Figures and Tables

**Table 1:** Characteristics of the young adults who participated in the study.

Participant	Sex	Age (Years)	Age at Parent’s Death (Years)	Deceased Parent’s Sex	Currently Living with
I	Female	21	17	Female	Father and sister
II	Male	19	14	Male	Mother and siblings
III	Male	24	19	Female	Father and sister
IV	Male	18	11	Female	Father and sister
V	Male	23	19	Male	Mother and siblings
VI	Female	20	13	Female	Father
VII	Female	19	15	Male	Mother
VIII	Female	18	16	Female	Independent
IX	Female	22	18	Male	Grandparents

**Table 2:** Categories and subcategories emerged by the formal analysis.

Categories	Subcategories
Anticipation of death as a process of emotional activation	Imminence of the parent’s death Activation of emotions and thoughts Closeness in the relationship Affective closure
Immediate emotional impact and suspension of one’s own experience at the moment of loss	Emotional response to the loss Displacement of one’s own emotions Emotional flattening Experience of death rituals within our culture Prolonged and persistent experience of the loss over time
Understanding death and loss	Sense of timelessness of the loss Meaning attributed to the death Loyalties toward the deceased parent Feeling of abandonment Internal phenomenology of the loss
The internal phenomenology of loss	Feeling unprotected Loneliness Yearning Disbelief or denial Confusion regarding what happened Loss as a turning point in life perception
Changes in life perception	Attribution of meaning to the loss Changes in life’s purpose or sense Acceptance of changes in the environment Reconfiguration of family and social relationships following the loss
Reconfiguring family and social relationships after loss	Changes in sibling relationships Changes in relationships with the surviving parent Changes in peer relationships Changes in family routines and roles Impact of the loss on personal development and self-construction
Impact on the construction of the self during adolescence	Self-awareness Rebelliousness Development of autonomy Empathy Search for emotional intimacy Representation of the deceased parent and quality of the maintained bond
Memories and connection with the deceased parent	Memories of shared routines Approach and interest toward the parent figure Recognition of the parent’s flaws within the bond Family and cultural worldview framing the experience of death
Family and cultural worldview framing the experience of death	Family values Family qualities Religious beliefs Death rituals Life lessons and re-signification of the experience over time
Re-signification and life lessons derived from the loss	Vocational interests influenced by the loss Connection with the social world Social values Symbolic continuity with the deceased parent

## Data Availability

Third-Party Data. Restrictions apply to the availability of these data. Data was obtained from anonymous informants and are available through the authors with the permission of the informants.
